# Retraction Note: Nuclear factor of activated T cells 5 maintained by Hotair suppression of miR-568 upregulates S100 calcium binding protein A4 to promote breast cancer metastasis

**DOI:** 10.1186/s13058-018-1021-z

**Published:** 2018-07-25

**Authors:** Jun-Tang Li, Li-Feng Wang, Ya-Li Zhao, Tao Yang, Wei Li, Jing Zhao, Feng Yu, Lei Wang, Yan-Ling Meng, Ning-Ning Liu, Xiao-Shan Zhu, Chun-Fang Gao, Lin-Tao Jia, An-Gang Yang

**Affiliations:** 1 State Key Laboratory of Cancer Biology, Department of Immunology, Fourth Military Medical University, Xi’an, 710032 Shaanxi China; 2 Institute of Anal-Colorectal Surgery, No. 150 Central Hospital of PLA, Luoyang, 471000 Henan China; 3 Department of Biochemistry and Molecular Biology, Fourth Military Medical University, Xi’an, 710032 Shaanxi China

## Retraction

The authors are retracting this article [1] after an investigation by the Ethics Committee of the Fourth Military Medical University (Xi’an, Shaanxi, China) of the following concerns that had been raised with respect to two of the figures:

Figure 1C, upper panel: areas of the image labelled as “Mock” and of that shown as “Scramble” are duplicated.

Figure 1D: the image labelled as “Scramble, 24 h” and that shown as “siNFAT5, 48 h” are duplicated.

Figure 3A: areas of the image labelled as “Invaded, siCtrl” and that shown as “Before Scraping, siS100A4” are duplicated.

## References

[1] Li J-T, Wang L-F, Zhao Y-L, Yang T, Li W, Zhao J (2014). Nuclear factor of activated T cells 5 maintained by Hotair suppression of miR-568 upregulates S100 calcium binding protein A4 to promote breast cancer metastasis. Breast Cancer Res.

